# Associations of eating speed with fat distribution and body shape vary in different age groups and obesity status

**DOI:** 10.1186/s12986-022-00698-w

**Published:** 2022-09-13

**Authors:** Saili Ni, Menghan Jia, Xuemiao Wang, Yun Hong, Xueyin Zhao, Liang Zhang, Yuan Ru, Fei Yang, Shankuan Zhu

**Affiliations:** 1 The Children’s Hospital, Zhejiang University School of Medicine, National Clinical Research Center for Child Health, Hangzhou, Zhejiang China; 2 Chronic Disease Research Institute, The Children’s Hospital, and National Clinical Research Center for Child Health, School of Public Health, School of Medicine, Zhejiang University, 866 Yu-hang-tang Road, Hangzhou, 310058 Zhejiang China; 3 Department of Nutrition and Food Hygiene, School of Public Health, Zhejiang University, Hangzhou, Zhejiang China; 4 Program in Public Health, University of California, Irvine, CA USA; 5 Department of Health, Lanxi, Zhejiang China

**Keywords:** Eating speed, Fat distribution, Body shape, Age, Obesity

## Abstract

**Background:**

Eating speed has been reported to be associated with energy intake, body weight, waist circumference (WC), and total body fat. However, no study has explored the association between eating speed and body fat distribution, especially its difference among different age or body mass index (BMI) groups.

**Methods:**

4770 participants aged 18–80 years were recruited from the baseline survey of the Lanxi Cohort Study. They were categorized into three groups according to meal duration. Linear regression analyses were performed among all participants and separately by age group and obesity status to evaluate the associations of WC and total and regional fat mass percentages (FM%) with eating speed.

**Results:**

After adjusting for confounding factors, eating slowly was significantly related to lower WC, lower total, trunk, and android FM%, lower android-to-gynoid fat mass ratio, and higher leg and gynoid FM%. After stratification by age or obesity status, the associations were especially prominent among participants aged 18–44 years or those with BMI < 24 kg/m^2^. No significant trends were found for participants aged 65–80 years or those who were overweight/obese.

**Conclusions:**

Eating slowly is closely related with better fat distribution among Chinese adults, especially for those aged 18–44 years and those with BMI < 24 kg/m^2^. If confirmed prospectively, it might be a potential efficient approach to improve fat distribution.

**Supplementary Information:**

The online version contains supplementary material available at 10.1186/s12986-022-00698-w.

## Background

The increasing global incidence of obesity has become a recognized public health problem, which results in a heightened risk of hypertension, dyslipidemia, diabetes, cardiovascular disease, gallbladder disease, and other disorders [1, 2]. In China, the prevalence of overweight and obesity have consistently increased over the past few decades, accounting for 32.3% and 6.2% of the adult population in 2016, respectively [3, 4], and these numbers will continue to increase with the development of social economy. Traditional indicators of obesity including body mass index (BMI) and waist circumference (WC). However, obesity has recently been defined as excessive fat accumulation and/or abnormal fat distribution in the body rather than simply body weight increase [5, 6]. Thus, total and regional fat percentages are considered more accurate indicators of obesity. Moreover, body fat distribution has also been reported to be more closely related to metabolic risks and diseases than traditional indicators of obesity [7–9]. Thus, it is crucial to seek a practical and effective strategy to prevent obesity, especially to promote body fat distribution.

Eating slowly has been reported as a simple and effective behavioral strategy to control energy intake and thus body weight [10, 11], and it is also associated with obesity [12, 13]. Moreover, some studies have focused on the relationship between eating speed and body shape, finding that eating more slowly is closely related to lower WC and lower total body fat percentage [12, 14, 15]. However, no previous study has explored the association between eating speed and body fat distribution. Whether eating slowly can improve body fat distribution is still unclear.

Additionally, although the positive association of eating quickly with increased BMI and obesity has been well documented among adults [13, 16, 17], adolescents, and school-aged children [18–20], some studies have indicated that eating behavior interventions may be more effective and have a longer impact among children and adolescents than among adults [21]. Some studies have also explored the influence of eating speed on energy intake or obesity-related chronic diseases such as non-alcoholic fatty liver disease (NAFLD) among different obesity status and found inconsistent results [9, 10]. These studies indicate that the associations between eating speed and obesity indexes might differ by individuals’ age or obesity status. However, studies specifically exploring the associations of eating speed with body shape and fat distribution among different age groups and obesity status are lacking.

Hence, the objectives of this study were to investigate whether eating speed is associated with body shape and fat distribution among Chinese adults and to further analyze the impact of age and obesity status on this association. We hypothesized that eating slowly may improve body fat distribution.

## Methods

### Participants

A total of 5 132 participants aged 18–80 years were recruited from the baseline survey of the Lanxi Cohort Study, which was conducted in urban and rural areas of Lanxi, Zhejiang Province, China, from June 2015 to August 2017. The Lanxi Cohort Study was established to systematically investigate the aetiology and interplay of body fat distribution and multiple factors with obesity and obesity-related non-communicable diseases in China [22]. It was approved by the Ethics Committee of the Zhejiang University School of Public Health. Written informed consent was obtained from each participant. Participants missing information from the whole-body dual-energy X-ray absorptiometry (DXA) measurements (n = 77), physical examination (n = 45), or questionnaires (n = 240) were excluded. A total of 4 770 participants were finally included in the analyses.

### Data collection

Participants were interviewed face-to-face by trained investigators to complete the questionnaires about demographic characteristics (age, sex, marital status and education level), lifestyle factors (smoking, drinking, type of meal, physical activity, and sleep quality) and medical history.

Anthropometric measurements were conducted by well-trained investigators according to standardized protocols [23]. With participants wearing light clothing and without shoes, weight (to 0.01 kg) and height (to 0.1 cm) were measured. BMI was calculated by dividing weight (in kilograms) by height (in meters) squared. WC was measured at the midpoint between the lowest costal margin and the iliac crest, with participant standing and at the end of an exhalation.

DXA scanning (software version 11.40.004; GE Lunar Prodigy, Madison, WI, USA) was used to measure total and regional fat mass (FM), including arms, trunk, android, gynoid and legs. For measures of the android region, the top of the pelvis was used as the lower boundary, and the upper boundary was above the pelvis line of demarcation at a position equivalent to 20% of the distance between the pelvis and the femoral neck. The lateral boundaries were the lines of the arms in a normal position during a whole-body scan. The gynoid region was defined by an upper boundary positioned below the pelvis cut line by 1.5 times the height of the android region. The lower boundary was positioned to equal twice the height of the android region. The lateral boundaries were the outer leg lines of demarcation [24]. DXA was calibrated daily using a standard phantom provided by manufacturer and was conducted by trained technicians according to a standard protocol [25].

### Total and regional body fat distribution

Total fat mass percentage (total FM%) was calculated by dividing total FM by total body mass. Regional fat mass percentage (regional FM%) was calculated by dividing regional FM by total FM. In addition, the android-to-gynoid fat mass ratio (AOI, android FM divided by gynoid FM) was calculated to assess the body FM distribution.

### Eating speed

Eating speed was measured by asking, “How long does it usually take you to have a meal: < 10 min, 10–19 min, 20–29 min, 30–39 min, 40–49 min, 50–59 min, or 60 min or more?” Because of the limited sample size, participants who answered 20–29, 30–39, 40–49, 50–59, or 60 min or more were combined into a category of ≥ 20 min. Participants were finally classified into three groups: < 10 min, 10–19 min, and ≥ 20 min.

### Covariates

Marital status was defined as “yes” or “no” [26]. Education level was categorized as “illiterate or primary school”, “middle school”, or “college or above” [27]. Smoking status was classified as “currently smoking” or “never smoked or quit smoking” [9, 14]. Participants who occasionally or frequently drank alcohol were defined as drinkers [14]. Type of meal was classified as “meat-based”, “balanced”, or “vegetable-based”. The International Physical Activity Questionnaire short form [28] was adopted to measure physical activity, which was leveled as low, moderate, or high [9, 14]. The Pittsburgh Sleep Quality Index (PSQI), which had been validated in the Chinese population and showed a high reliability coefficient and test–retest reliability, was used to measure sleep quality [14, 29]. PSQI scores ranged from 0 to 21, with higher scores indicating worse sleep quality. Besides, age, sex and BMI were also listed as covariates [9, 14]. Study cohorts were categorized as urban or rural according to the areas from which the participants were recruited. Detailed comparisons between participants from rural and urban areas are showed in Additional file 1: Table S1.

### Statistical analyses

Variables are presented as means and standard deviations for continuous variables and as counts and percentages for categorical variables. Differences between eating speed groups were tested using analysis of variance and chi-square tests.

Multivariable linear regression analyses were used to evaluate the associations of WC and total and regional FM% with eating speed. Interactions were found between age or obesity status and eating speed (*P* values for interactions between age and eating speed are 0.0256, 0.1459, 0.3524, 0.0045, 0.0009, 0.0016, 0.0012, 0.0048, and for interactions between obesity status and eating speed are 0.3568, 0.9662, 0.0880, 0.0176, 0.0218, 0.1017, 0.0075, 0.0016 for WC, total FM%, arms FM%, trunk FM%, android FM%, AOI, gynoid FM% and legs FM%, respectively), and models were first estimated for all participants and then separately for different age (relative young: 18–44 years; middle-aged: 45–64 years; relative elder: ≥ 65 years) [30] and obesity status (BMI ≥ 24 kg/m^2^ was considered as overweight/obese) [31, 32]. All models were adjusted for study cohort, age, sex, marital status, education level, smoking, drinking, type of meal, physical activity level, sleep quality and BMI. To exclude the impact of diseases and emotional stress, sensitivity analyses were performed, with models being additionally adjusted for chronical diseases (including cardiovascular diseases, metabolic diseases, osteoarticular diseases and cancers) and the score of the World Health Organization Well-Being Index-5 (WHO-5), respectively [33]. Models adjusted for the score of WHO-5 were performed among urban participants only, as rural participants had not collected information on it. Moreover, interactive analyses between covariates and eating speed with respect to fat distribution indexes were also conducted. In addition, multivariable linear regression analyses stratified by covariates were carried out to investigate the robustness and heterogeneity of the associations. A cutoff score of 7 or higher was used to screen for poor sleep quality in analyses stratified by PSQI scores [34].

Statistical analyses were performed using SAS, Version 9.2 (SAS Institute, Inc., Cary, NC, USA). *P* < 0.05 (two-tailed) was considered statistically significant.

## Results

Table 1 shows the baseline characteristics of the participants by eating speed groups, revealing differences among groups in cohort, sex, educational level, smoking, drinking, type of meal, physical activity, sleep quality, BMI, WC, and total and regional FM%. Compared with participants reporting a meal duration of < 10 min, BMI, WC, trunk FM%, android FM%, and AOI were lower in the other two groups while gynoid FM% and leg FM% were higher (*P* < 0.05).

**Table 1:** Baseline characteristics of the participants (n = 4770)

Variables	Meal duration			P*
	< 10 min (n = 1569)	10–19 min (n = 2328)	≥ 20 min (n = 873)	
Study cohort (urban), n (%)	946 (60.29)^§^	1574 (67.61)	575 (65.86)^†^	< 0.0001
Age, y	53.42 ± 11.79	52.89 ± 12.92	53.54 ± 13.42	0.2885
Men, n (%)	644 (41.05)^§^	830 (35.65)	346 (39.63)	0.0019
Married, n (%)	1447 (92.22)	2103 (90.34)	792 (90.72)	0.1213
Education level, n (%)				0.0005
Illiteracy or primary school	678 (43.21)	894 (38.40)	315 (36.08)	
Middle school	753 (47.99)	1158 (49.74)	463 (53.04)	
College school or above	138 (8.80)	276 (11.86)	95 (10.88)	
Smoker, n (%)	304 (19.38)^§^	315 (13.53)^#^	150 (17.18)	< 0.0001
Drinker, n (%)	688 (43.85)	956 (41.07) ^#^	487 (55.78) ^†^	< 0.0001
Type of meal, n (%)				0.0005
Meat-based	119 (7.58)	161 (6.92)	92 (10.54)	
Balanced	990 (63.10)	1570 (67.44)	566 (64.83)	
Vegetable-based	460 (29.32)	597 (25.64)	215 (24.63)	
Physical activity, n (%)				< 0.0001
Low	532 (33.91)	679 (29.17)	259 (29.67)	
Middle	598 (38.11)	1051 (45.15)	352 (40.32)	
High	439 (27.98)	598 (25.69)	262 (30.01)	
Sleep quality	5.48 ± 2.55^§^	5.25 ± 2.59	5.43 ± 2.57	0.018
BMI, kg/m2	23.70 ± 3.13^§^	23.36 ± 3.20	23.07 ± 3.28^†^	< 0.0001
WC, cm	83.79 ± 9.36^§^	82.31 ± 9.64	81.78 ± 9.91^†^	< 0.0001
Fat mass, %				
Total	29.06 ± 7.87	29.07 ± 7.56^#^	28.05 ± 7.99^†^	0.0021
Arms	9.94 ± 1.71	10.02 ± 1.67^#^	9.81 ± 1.72	0.0102
Trunk	59.13 ± 5.53^§^	58.25 ± 5.56	58.07 ± 6.10^†^	< 0.0001
Android	10.94 ± 1.51^§^	10.71 ± 1.58	10.71 ± 1.71^†^	< 0.0001
Gynoid	16.48 ± 2.94^§^	16.92 ± 2.97	17.15 ± 3.33^†^	< 0.0001
Legs	26.77 ± 5.06^§^	27.52 ± 5.18	27.84 ± 5.73^†^	< 0.0001
AOI	0.70 ± 0.19^§^	0.66 ± 0.19	0.66 ± 0.21^†^	< 0.0001

Data are mean ± SD, n (%) unless otherwise indicated

Abbreviations: BMI, body mass index; WC, waist circumference; AOI, android-to-dynoid fat mass ratio

P*Analysis of variance or Chi-square tests among participants in three groups

^§^*P* < 0.05, significant difference between participants in group 1 and group 2

^#^*P* < 0.05, significant difference between participants in group 2 and group 3

^†^*P* < 0.05, significant difference between participants in group 1 and group 3

The adjusted associations between eating speed and the body fat distribution indexes (WC, total and regional FM%) are shown in Table 2. As meal duration increased, WC, total FM%, trunk FM%, android FM%, and AOI decreased, whereas leg FM% and gynoid FM% increased. Additionally, compared with the group whose meal duration was < 10 min, the other two groups had lower WC, total FM%, trunk FM%, and AOI and higher leg FM% and gynoid FM%. Participants whose meal duration was ≥ 20 min also had lower android FM% than those whose meal duration was < 10 min.

**Table 2:** Associations between eating speed and body fat distribution (n = 4770)

Variables	Meal duration						P* for trend
	< 10 min (n = 1569)		10–19 min (n = 2328)		≥ 20 min (n = 873)		
	β^#^	*P*	β^#^	*P*	β^#^	*P*	
WC	–	–	− 0.359	0.0151	− 0.402	0.0357	0.0168
Total FM%	–	–	− 0.314	0.0144	− 0.419	0.0118	0.0058
Arms FM%	–	–	− 0.006	0.8952	− 0.091	0.1132	0.1579
Trunk FM%	–	–	− 0.298	0.0385	− 0.646	0.0005	0.0005
Android FM%	–	–	− 0.065	0.1167	− 0.145	0.0067	0.0064
AOI	–	–	− 0.011	0.0294	− 0.017	0.0078	0.0048
Gynoid FM%	–	–	0.155	0.0309	0.349	0.0002	0.0002
Legs FM%	–	–	0.279	0.0479	0.717	< 0.0001	0.0001

Models were adjusted for study cohort, age, sex, education level, marital status, smoking, drinking, type of meal, physical activity level, sleep quality and body mass index

Abbreviations: WC, waist circumference; FM%, fat mass percentage; AOI, android-to-dynoid fat mass ratio

β^#^: Unstandardized regression coefficient

P*: Trend tests among participants in three groups

After stratification by age, associations between eating speed and body fat distribution were further analyzed (Table 3). Among participants in the relative young group, WC, trunk FM%, android FM%, and AOI tended to decrease while leg FM% and gynoid FM% tended to increase as meal duration increased (all *P* for trend < 0.05). Among participants aged 45 to 64 years, a significant trend was found only in total FM%. No significant associations between eating speed and body fat distribution were found in relative elder group.

**Table 3:** Associations between eating speed and body fat distribution among different age groups

Variables	18 ≤ age < 45 (n = 1123)							45 ≤ age < 65 (n = 2681)							65 ≤ age ≤ 80 (n = 966)						
	Meal duration < 10 min (n = 336)		10 ≤ meal duration < 20 min (n = 567)		Meal duration ≥ 20 min (n = 220)		P* for trend	Meal duration < 10 min (n = 945)		10 ≤ meal duration < 20 min (n = 1298)		Meal duration ≥ 20 min (n = 438)		P* for trend	Meal duration < 10 min (n = 288)		10 ≤ meal duration < 20 min (n = 463)		Meal duration ≥ 20 min (n = 215)		P* for trend
	β^#^	*P*	β^#^	*P*	β^#^	*P*		β^#^	*P*	β^#^	*P*	β^#^	*P*		β^#^	*P*	β^#^	*P*	β^#^	*P*	
WC	–	–	− 0.368	0.2305	− 0.877	0.0222	0.0230	–	–	− 0.509	0.0078	− 0.148	0.5705	0.2032	–	–	0.389	0.2604	0.124	0.7664	0.6810
Total FM%	–	–	− 0.417	0.1416	− 0.602	0.0893	0.0757	–	–	− 0.301	0.0667	− 0.504	0.0242	0.0150	–	–	− 0.228	0.4391	− 0.069	0.8453	0.7840
Arms FM%	–	–	0.010	0.9054	− 0.143	0.1859	0.2336	–	–	0.001	0.9838	0.023	0.7708	0.8032	–	–	− 0.011	0.9198	− 0.113	0.3868	0.4121
Trunk FM%	–	–	− 0.347	0.2034	− 0.734	0.0312	0.0305	–	–	− 0.228	0.2061	− 0.315	0.1991	0.1465	–	–	− 0.079	0.8351	− 0.412	0.3701	0.3887
Android FM%	–	–	− 0.090	0.2548	− 0.287	0.0036	0.0046	–	–	− 0.079	0.1206	0.000	0.9990	0.6415	–	–	0.052	0.6399	− 0.099	0.4592	0.5252
AOI	–	–	− 0.016	0.0479	− 0.033	0.0013	0.0012	–	–	− 0.010	0.1118	− 0.006	0.5085	0.3031	–	–	0.002	0.8669	− 0.006	0.7089	0.7423
Gynoid FM%	–	–	0.267	0.0576	0.623	0.0004	0.0004	–	–	0.124	0.1728	0.172	0.1662	0.1175	–	–	− 0.014	0.9386	0.088	0.6987	0.7246
Legs FM%	–	–	0.335	0.2198	0.877	0.0102	0.0113	–	–	0.192	0.2779	0.259	0.2835	0.2219	–	–	0.093	0.7973	0.565	0.1961	0.2158

Models were adjusted for study cohort, age, sex, education level, marital status, smoking, drinking, type of meal, physical activity level, sleep quality and body mass index

Abbreviations: WC, waist circumference; FM%, fat mass percentage; AOI, android-to-dynoid fat mass ratio

β^#^Unstandardized regression coefficient

P*Trend tests among participants in three eating speed subgroups in each age group

We also investigated the relationships between eating speed and body fat distribution among different obesity status (Table 4). In participants with BMI < 24 kg/m^2^, eating slowly was associated with lower WC, total FM%, arm FM%, trunk FM%, android FM%, and AOI and higher leg FM% and gynoid FM%. Significant differences of all fat mass indexes were found between participants with meal duration of ≥ 20 min and those with meal duration of < 10 min. Between participants whose meal duration from 10 to 19 min and whose meal duration < 10 min, significant differences were only found among WC and total FM%. Among participants with BMI ≥ 24 kg/m2, no significant trends were found in all index. Additionally, those with a meal duration of 10 to 19 min had lower trunk FM%, AOI and higher gynoid FM%, compared with those with a meal duration of < 10 min.

**Table 4:** Associations between eating speed and body fat distribution among different obesity status

Variables	BMI < 24 kg/m^2^ (n = 2876)							BMI ≥ 24 kg/m^2^ (n = 1894)						
	Meal duration < 10 min (n = 880)		10 min ≤ Meal duration < 20 min (n = 1450)		Meal duration ≥ 20 min (n = 546)		P* for trend	Meal duration < 10 min (n = 689)		10 min ≤ Meal duration < 20 min (n = 878)		Meal duration ≥ 20 min (n = 327)		P* for trend
	β^#^	*P*	β^#^	*P*	β^#^	*P*		β^#^	*P*	β^#^	*P*	β^#^	*P*	
WC	–	–	− 0.525	0.0035	− 0.316	0.1695	0.0729	–	–	− 0.075	0.7602	− 0.352	0.2798	0.3156
Total FM%	–	–	− 0.408	0.0149	− 0.441	0.0398	0.0211	–	–	− 0.178	0.3291	− 0.093	0.6990	0.5489
Arms FM%	–	–	− 0.037	0.5146	− 0.198	0.0061	0.0102	–	–	0.008	0.9082	0.077	0.4133	0.4669
Trunk FM%	–	–	− 0.235	0.2261	− 0.586	0.0184	0.0196	–	–	− 0.393	0.0365	− 0.403	0.1045	0.0500
Android FM%	–	–	− 0.053	0.3683	− 0.160	0.0335	0.0387	–	–	− 0.087	0.1009	− 0.049	0.4830	0.2931
AOI	–	–	− 0.005	0.3952	− 0.019	0.0226	0.0281	–	–	− 0.019	0.0075	− 0.006	0.5558	0.2144
Gynoid FM%	–	–	0.079	0.4299	0.364	0.0047	0.0076	–	–	0.265	0.0012	0.108	0.3159	0.0803
Legs FM%	–	–	0.227	0.2411	0.808	0.0011	0.0018	–	–	0.382	0.0375	0.296	0.2221	0.1064

Models were adjusted for study cohort, age, sex, education level, marital status, smoking, drinking, type of meal, physical activity level, sleep quality and body mass index

Abbreviations: WC, waist circumference; FM%, fat mass percentage; AOI, android-to-dynoid fat mass ratio; BMI, body mass index

β^#^: Unstandardized regression coefficient

P*: Trend tests among participants in three eating speed subgroups in different obesity status

As for the sensitivity analyses, all results remained similar when being additionally adjusted for chronical diseases or the score of WHO-5 (Additional file 1: Table S2, Table S3). Interactions were found between marital status, education level, smoking or drinking status and eating speed, while no interactions were found between sex, type of meal, physical activity, sleep quality or study cohort and eating speed for WC and for total and regional FM% (Additional file 1: Table S4). The results kept stable after stratified by those covariates and the direction of regression coefficients remained fairly consistent (Additional file 1: Table S5-Table S13).

## Discussion

This study found that eating speed was closely associated with body shape and fat distribution among Chinese adults. Participants who had a slower eating speed had smaller WC, total FM%, trunk FM%, android FM%, and AOI, but higher leg FM% and gynoid FM%. After being analyzed separately by age and obesity status, the associations of WC and total and regional FM% with eating speed were especially prominent among participants aged 18–44 years and those with BMI < 24 kg/m^2^.

Previous studies have well documented that eating speed is closely related to body weight and the prevalence of obesity [11–13, 16, 18]. Eating slowly encourages lower energy density and energy intake, which may play a major role in preventing overweight and/or obesity [35]. Some studies have also indicated that eating slowly is related to lower WC and total FM% measured by bioimpedance analysis [12, 14, 15], which is consistent with our results. Body fat parameters, compared with traditional indicators such as BMI, can better reflect obesity status of individuals [6–8]. Moreover, it has been suggested that the location of fat tissue can influence its autocrine, paracrine, and endocrine effects and that the location of fat tissue can explain the relatively higher risk of metabolic disease better than can total fat [36, 37]. However, previous studies have used only total FM and WC as parameters of body composition and shape, and there have been no previous studies exploring the association between eating speed and body fat distribution. Our study firstly analyzed the associations between eating speed and regional FM% in various sites, finding that eating more slowly is related with lower trunk FM%, android FM% (around the trunk/abdomen area), and AOI and higher leg FM% and gynoid FM% (located in the hip and thigh). The strong associations of accumulated truncal and abdominal fat with adverse metabolic profiles and the increasing prevalence of metabolic diseases have been well documented [38–40]. It has been reported that both dysfunctional abdominal subcutaneous adipose tissue and visceral adipose tissue accumulation can lead to inflammatory dysregulation and adverse metabolic effects like insulin resistance or dyslipidemia. However, lower-body fat storage is indicated to protect other tissues from lipotoxicity caused by ectopic fat deposition [41, 42]. Previous studies have also found negative correlations between gynoid or leg FM% and metabolic risk factors such as blood pressure, fasting plasma glucose level, and lipid profiles, indicating that lower-body fat accumulation might have favorable effects on the risk of metabolic diseases [38, 43, 44]. Besides, leg fat mass was also found to be negatively associated with risk of cardiometabolic disorders and type 2 diabetes mellitus [45, 46]. AOI, which is calculated by dividing android FM by gynoid FM, has been suggested to be a better indicator of central fat distribution than waist-to-thigh or waist-to-hip ratio [47]. It has also been found to be related with triglyceride/high-density lipoprotein cholesterol ratio and is considered to be an important predictor of cardiovascular diseases [43, 48]. The results of the present study indicate that eating slowly may not only associated with reduced total FM%, but also with better whole-body fat distribution, which may be especially important for preventing obesity and metabolic diseases [36, 37, 43].

When analyzing the participants separately by age group, we found that associations of WC and total and regional FM% with eating speed varied by age group. These effects were somewhat more robust among participants aged 18–44 years and then weakened with aging. The prevalence of many chronic diseases, such as cardiovascular diseases, metabolic diseases, osteoarticular diseases and cancers, were higher among relative elder participants (Additional file 1: Table S14), which might influence the impact of eating speed on body fat distribution. However, after additionally adjusted for those diseases, the results remained similar among all three age groups as associations between eating speed and fat distribution were still more prominent among participants aged 18–44 years, indicating that association between eating speed and fat distribution might be influenced by age (Additional file 1: Table S15). It has been well documented that, with increasing age, body fat becomes centralized and is redistributed from subcutaneous to visceral depots, even among healthy people [49, 50]. Aging-associated changes in fat distribution occur irrespective of sex or race and are accompanied by an increased risk of multiple sclerosis and adipose tissue chronic inflammation, and decreased proliferation and differentiation of preadipocytes [51]. Therefore, differences in eating speed may not be associated with significant variation in body shape and fat distribution among older participants. However, the close associations of body shape and fat distribution with eating speed among younger adults are particularly important as promotion of their body shape and fat distribution can bring huge benefits in later life. If the effect of eating slowly for promoting body fat distribution are proved with further prospective studies, eating slowly will be a simple and efficient method to promote long-term health for young people.

Obesity status has also been suggested to be related to the impact of eating speed. Meena Shah et al. [10] found that eating slowly significantly lowered meal energy intake among people of normal weight, but not among those who were overweight/obese. In a recent study, Saehyun Lee et al. [9] found that self-reported eating speed is associated with an increased risk of the presence and grade of NAFLD among Korean adults, especially those with BMI < 25 kg/m^2^. They suggested that the effect of obesity on NAFLD may overwhelm any effect of eating speed on it in obese group. These studies may partly explain the results of our BMI-stratified analyses. It is notable that the associations of WC and total and regional FM% with eating speed showed less significance in the overweight/obese group. Trend test even showed no significance among all indexes. Obesity has complex causes, which include heredity, environmental factors, and behavior [52]. Compared with some of the other risk factors, the impact of eating speed on WC and total and regional FM% might be limited for overweight/obese participants. Moreover, as BMI increased, WC and body fat also increased, whereas body fat distribution worsened and gradually reached a relatively steady state [53, 54]. Thus, WC and total and regional FM% may be more difficult to change among overweight/obese people than among those of normal weight. As for the results in the normal group, our study indicated that total and regional FM% varied across different eating speed groups even among participants all with normal BMI. Considering the closer relationship between the risk of metabolic diseases and total and regional fat mass than that between the risk of metabolic diseases and total and BMI, our findings are notable for normal-BMI people [7–9, 36–40]. If our results are confirmed by prospective studies, eating slowly might become a potential efficient intervention to improve body shape and fat distribution among people of normal weight. However, for overweight/obese people, more powerful and comprehensive strategies should be proposed.

This study had several strengths. First, our study was based on a large study population, making our results more reliable. Second, a series of important potential confounding factors, such as age, sex, education level, physical activity, type of meal, and sleep quality were adjusted [51], making the results more objective. However, it should still be noted that the associations of WC and total and regional FM% with eating speed could be caused by unmeasured confounding factors associated with both fat mass and eating behavior. In addition, our study used total and regional FM% measured by DXA, which is considered more reliable than bioimpedance analysis, as indexes of body shape and fat mass. It has been documented that android FM% and AOI can better reflect abnormal fat accumulation and central adiposity than can traditional indexes such as WC [55]. However, only a few studies have previously taken fat mass into consideration, using only total FM% as measured by bioimpedance analysis as an index [12]. Our study was the first to analyze the relationship between eating speed and fat distribution, and we further analyzed the association of this relationship with individuals’ age and obesity status. Our results enhance the understanding of the associations of fat distribution and obesity with eating speed.

Several limitations of this study should also be noted. First, because of the cross-sectional design of the study, causal relationships between eating speed and body shape and fat distribution cannot be inferred. Second, we used self-reported meal duration to reflect eating speed and the options were spread too far apart, which may affect the accuracy of it. More appropriate method should be used in the future to collect more accurate information on eating speed. Furthermore, we do not have information on total energy intake. However, we found that associations between eating speed and body fat distribution indexes are still significant after adjusting for BMI, which has been reported to be closely related with total energy intake [56]. We also adjusted for type of meal which can somewhat reflect participants’ eating habits. Additionally, hormones such as peptide YY, glucagon-like peptide-1, and cholecystokinin, which have been reported to be related to the mechanism underlying the influence of eating speed [17, 57], should be measured in the future to explore how eating speed affects body shape and fat distribution.

## Conclusion

In conclusion, eating speed is associated with WC and total and regional FM% among Chinese adults, especially those aged 18–44 years and those with BMI < 24 kg/m^2^. Eating slowly is closely associated with better fat distribution among relative young and normal-weight individuals. However, for older or overweight/obese individuals, more powerful and comprehensive strategies should be used. Prospective cohort studies and intervention trials should be conducted in the future to further analyze the association between eating speed and fat distribution and to clarify the mechanism underlying this association.

## Supplementary Information

**Additional file 1.** Sensitivity and interaction analyses.

## Data Availability

The datasets generated during and/or analyzed during the current study are not publicly available due to participants’ privacy but are available from the corresponding author on reasonable request.

## Abbreviations

AOI: Android-to-gynoid fat mass ratio
BMI: Body mass index
DXA: Dual-energy X-ray absorptiometry
FM: Fat mass
FM%: Fat mass percentages
NAFLD: Non-alcoholic fatty liver disease
PSQI: Pittsburgh Sleep Quality Index
WC: Waist circumference
WHO-5: The World Health Organization Well-Being Index-5
