# Supplementary material for: Associations of eating speed with fat distribution and body shape vary in different age groups and obesity status
Source: Nutr Metab (Lond). 2022 Sep 13;19:63. doi: 10.1186/s12986-022-00698-w (PMC9469611; doi:10.1186/s12986-022-00698-w)
Supplement: Supplementary file 1 — Additional file 1. Sensitivity and interaction analyses. [file 12986_2022_698_MOESM1_ESM.docx]

**Table S1. Baseline characteristics among participants from different areas**

| **Variables** | **Study cohort** | | ***P**** |
| --- | --- | --- | --- |
|  | **urban (n=3095)** | **rural (n=1675)** |  |
| Meal duration, n(%) |  |  | <0.0001 |
| <10 min | 946 (30.57) | 623 (37.19) |  |
| 10-19 min | 1574 (50.86) | 754 (45.01) |  |
| ≥20 min | 575 (18.58) | 298 (17.79) |  |
| Age, y | 52.59 ± 13.00 | 54.29 ± 11.90 | <0.0001 |
| Men, n(%) | 1151 (37.19) § | 669 (39.94) | 0.0619 |
| Married, n(%) | 2769 (89.47) | 1573 (93.91) | <0.0001 |
| Education level, n(%) |  |  | <0.0001 |
| Illiteracy or primary school | 942 (30.44) | 945 (56.42) |  |
| Middle school | 1703 (55.02) | 671 (40.06) |  |
| College school or above | 450 (14.54) | 59 (3.52) |  |
| Smoker, n(%) | 488 (15.77) | 281 (16.78) | 0.3658 |
| Drinker, n(%) | 1404 (45.36) | 727 (43.4) | 0.1936 |
| Type of meal, n(%) |  |  | 0.0334 |
| Meat-based | 247 (7.98) | 125 (7.46) |  |
| Balanced | 1988 (64.23) | 1138 (67.94) |  |
| Vegetable-based | 860 (27.79) | 412 (24.6) |  |
| Physical activity, n(%) |  |  | <0.0001 |
| Low | 823 (26.59) | 647 (38.63) |  |
| Middle | 1657 (53.54) | 344 (20.54) |  |
| High | 615 (19.87) | 684 (40.84) |  |
| Sleep quality | 4.83 ± 2.77 | 6.33 ± 1.80 | <0.0001 |
| BMI, kg/m2 | 23.34 ± 3.19 | 23.56 ± 3.20 | 0.0207 |
| WC, cm | 82.47 ± 9.64 | 83.11 ± 9.59 | 0.0281 |
| Fat mass, % |  |  |  |
| Total | 29.62 ± 7.48 | 27.51 ± 8.05 | <0.0001 |
| Arms | 10.03 ± 1.72 | 9.82 ± 1.64 | <0.0001 |
| Trunk | 58.33 ± 5.59 | 58.84 ± 5.79 | 0.0031 |
| Android | 10.69 ± 1.57 | 10.97 ± 1.60 | <0.0001 |
| Gynoid | 16.89 ± 3.03 | 16.68 ± 3.04 | 0.0263 |
| Legs | 27.47 ± 5.25 | 27.08 ± 5.28 | 0.0139 |
| AOI | 0.66 ± 0.19 | 0.69 ± 0.20 | <0.0001 |

Data are mean ± SD, n (%) unless otherwise indicated.

Abbreviations: BMI, body mass index; WC, waist circumference; AOI, android-to-dynoid fat mass ratio.

*P**: Analysis of variance or Chi-square tests among participants from different areas.

**Table S2. Associations between eating speed and body fat distribution (additionally adjusted for** **chronic diseases)**

| **Variables** | **Meal duration** | | | | | | | | | | | | ***P** for trend** |
| --- | --- | --- | --- | --- | --- | --- | --- | --- | --- | --- | --- | --- | --- |
|  | **<10 min (n=1569)** | | | | **10-19 min (n=2328)** | | | | **≥20 min (n=873)** | | | |  |
|  | **β^#^** | | | ***P*** | **β^#^** | | ***P*** | | **β^#^** | | ***P*** | |  |
| WC | - | - | -0.364 | | | 0.0135 | | -0.412 | | 0.0309 | | 0.0143 | |
| Total FM% | - | - | -0.315 | | | 0.0142 | | -0.421 | | 0.0115 | | 0.0056 | |
| Arms FM% | - | - | -0.007 | | | 0.8761 | | -0.093 | | 0.1041 | | 0.1459 | |
| Trunk FM% | - | - | -0.304 | | | 0.0337 | | -0.659 | | 0.0004 | | 0.0003 | |
| Android FM% | - | - | -0.066 | | | 0.1073 | | -0.149 | | 0.0055 | | 0.0052 | |
| AOI | - | - | -0.011 | | | 0.0245 | | -0.017 | | 0.0056 | | 0.0033 | |
| Gynoid FM% | - | - | 0.159 | | | 0.0257 | | 0.356 | | 0.0001 | | 0.0001 | |
| Legs FM% | - | - | 0.286 | | | 0.0409 | | 0.731 | | <0.0001 | | <0.0001 | |

Models were adjusted for chronic diseases (including cardiovascular diseases, metabolic diseases, osteoarticular diseases and cancers), study cohort, age, sex, education level, marital status, smoking, drinking, type of meal, physical activity level, sleep quality and body mass index.

Abbreviations: WC, waist circumference; FM%, fat mass percentage; AOI, android-to-dynoid fat mass ratio.

β**^#^**: Unstandardized regression coefficient.

*P**: Trend tests among participants in three groups.

**Table S3. Associations between eating speed and body fat distribution among urban participants (n=3095)**

| **Variables** | **Meal duration** | | | | | | ***P** for trend** |
| --- | --- | --- | --- | --- | --- | --- | --- |
|  | **<10 min (n=946)** | | **10-19 min (n=1574)** | | **≥20 min (n=575)** | |  |
|  | **β^#^** | ***P*** | **β^#^** | ***P*** | **β^#^** | ***P*** |  |
| WC | - | - | -0.331 | 0.0665 | -0.418 | 0.0713 | 0.0489 |
| Total FM% | - | - | -0.375 | 0.0210 | -0.332 | 0.1112 | 0.0604 |
| Arms FM% | - | - | -0.049 | 0.3868 | -0.128 | 0.0803 | 0.0846 |
| Trunk FM% | - | - | -0.252 | 0.1563 | -0.669 | 0.0035 | 0.0040 |
| Android FM% | - | - | -0.083 | 0.1071 | -0.150 | 0.0238 | 0.0198 |
| AOI | - | - | -0.009 | 0.1446 | -0.013 | 0.1043 | 0.0828 |
| Gynoid FM% | - | - | 0.138 | 0.1240 | 0.348 | 0.0026 | 0.0029 |
| Legs FM% | - | - | 0.268 | 0.1300 | 0.756 | 0.0009 | 0.0012 |

Models were adjusted for the score of the World Health Organization Well-Being Index-5, age, sex, education level, marital status, smoking, drinking, type of meal, physical activity level, sleep quality and body mass index.

Abbreviations: WC, waist circumference; FM%, fat mass percentage; AOI, android-to-dynoid fat mass ratio.

β**^#^**: Unstandardized regression coefficient.

*P**: Trend tests among participants in three groups.

**Table S4. Interactive analyses among covariates and eating speed with respect to fat distribution indexes**

| **Variables** | ***P** for interactive analysis** | | | | | | | | |
| --- | --- | --- | --- | --- | --- | --- | --- | --- | --- |
|  | **Study cohort** | **Sex** | **Type of meal** | **Physical activity** | **Sleep quality** | **Marital status** | **Education level** | **Smoking** | **Drinking** |
| WC | 0.5008 | 0.0704 | 0.1711 | 0.2816 | 0.6613 | 0.0021 | 0.0816 | 0.0036 | 0.1157 |
| Total FM% | 0.4668 | 0.7510 | 0.7469 | 0.1847 | 0.0731 | 0.5953 | 0.0674 | 0.2614 | 0.1783 |
| Arms FM% | 0.0924 | 0.3468 | 0.8541 | 0.4432 | 0.3086 | 0.4789 | 0.7952 | 0.3005 | 0.8099 |
| Trunk FM% | 0.9900 | 0.2085 | 0.4867 | 0.2823 | 0.2552 | 0.0122 | 0.0006 | 0.0250 | 0.0605 |
| Android FM% | 0.8020 | 0.2494 | 0.9069 | 0.7749 | 0.1030 | 0.0260 | 0.0003 | 0.0044 | 0.0480 |
| AOI | 0.6139 | 0.3521 | 0.4659 | 0.4630 | 0.3978 | 0.0846 | 0.0059 | 0.0056 | 0.0440 |
| Gynoid FM% | 0.6890 | 0.0616 | 0.2396 | 0.3089 | 0.3931 | 0.0630 | 0.0018 | 0.0016 | 0.1682 |
| Legs FM% | 0.7899 | 0.1183 | 0.6002 | 0.3251 | 0.0802 | 0.0254 | 0.0006 | 0.0133 | 0.0499 |

Abbreviations: WC, waist circumference; FM%, fat mass percentage; AOI, android-to-dynoid fat mass ratio.

*P**: Interactive analyses between each covariate and eating speed with respect to fat distribution indexes.

**Table S5. Associations between eating speed and body fat distribution among different marital status**

| **Variables** | **Unmarried (n=428)** | | | | | | | **Married (n=4342)** | | | | | | |
| --- | --- | --- | --- | --- | --- | --- | --- | --- | --- | --- | --- | --- | --- | --- |
|  | **Meal duration<10 min (n=122)** | | **10≤meal duration<20 min (n=225)** | | **Meal duration≥20 min (n=81)** | | ***P** for trend** | **Meal duration<10 min (n=1447)** | | **10≤meal duration<20 min (n=2103)** | | **Meal duration≥20 min (n=792)** | | ***P** for trend** |
|  | **β^#^** | ***P*** | **β^#^** | ***P*** | **β^#^** | ***P*** |  | **β^#^** | ***P*** | **β^#^** | ***P*** | **β^#^** | ***P*** |  |
| WC | - | - | -0.623 | 0.2408 | -0.809 | 0.2367 | 0.2055 | - | - | -0.329 | 0.0327 | -0.385 | 0.0538 | 0.0292 |
| Total FM% | - | - | -0.702 | 0.1644 | -0.005 | 0.9938 | 0.8099 | - | - | -0.279 | 0.0353 | -0.479 | 0.0052 | 0.0034 |
| Arms FM% | - | - | 0.332 | 0.0348 | 0.066 | 0.7420 | 0.5159 | - | - | -0.033 | 0.4711 | -0.088 | 0.1444 | 0.1499 |
| Trunk FM% | - | - | -0.667 | 0.1865 | -1.064 | 0.1018 | 0.0892 | - | - | -0.245 | 0.1022 | -0.633 | 0.0011 | 0.0013 |
| Android FM% | - | - | 0.002 | 0.9869 | -0.250 | 0.1891 | 0.2371 | - | - | -0.070 | 0.1052 | -0.136 | 0.0148 | 0.0123 |
| AOI | - | - | -0.003 | 0.8477 | -0.022 | 0.2975 | 0.3305 | - | - | -0.011 | 0.0295 | -0.018 | 0.0080 | 0.0048 |
| Gynoid FM% | - | - | 0.218 | 0.3990 | 0.396 | 0.2338 | 0.2234 | - | - | 0.146 | 0.0519 | 0.361 | 0.0002 | 0.0002 |
| Legs FM% | - | - | 0.290 | 0.5761 | 0.924 | 0.1670 | 0.1795 | - | - | 0.257 | 0.0792 | 0.702 | 0.0002 | 0.0003 |

Models were adjusted for study cohort, age, sex, education level, smoking, drinking, type of meal, physical activity level, sleep quality and body mass index.

Abbreviations: WC, waist circumference; FM%, fat mass percentage; AOI, android-to-dynoid fat mass ratio.

β**^#^**: Unstandardized regression coefficient.

*P**: Trend tests among participants in three eating speed subgroups in different marital status.

**Table S6. Associations between eating speed and body fat distribution among different education levels**

| **Variables** | **Illiterate or primary school (n=1887)** | | | | | | | **Middle school (n=2374)** | | | | | | | **College or above (n=509)** | | | | | | | |
| --- | --- | --- | --- | --- | --- | --- | --- | --- | --- | --- | --- | --- | --- | --- | --- | --- | --- | --- | --- | --- | --- | --- |
|  | **Meal duration<10 min (n=678)** | | **10≤meal duration<20 min (n=894)** | | **Meal duration≥20 min (n=315)** | | ***P** for trend** | **Meal duration<10 min (n=753)** | | **10≤meal duration<20 min (n=1158)** | | **Meal duration≥20 min (n=463)** | | ***P** for trend** | **Meal duration <10 min (n=138)** | | **10≤meal duration<20 min (n=276)** | | **Meal duration≥20 min (n=95)** | | ***P** for trend** | |
|  | **β^#^** | ***P*** | **β^#^** | ***P*** | **β^#^** | ***P*** |  | **β^#^** | ***P*** | **β^#^** | ***P*** | **β^#^** | ***P*** |  | **β^#^** | ***P*** | **β^#^** | ***P*** | **β^#^** | ***P*** |  |  |
| WC | - | - | -0.234 | 0.3164 | -0.328 | 0.2951 | 0.2413 | - | - | -0.380 | 0.0654 | -0.635 | 0.0148 | 0.0113 | - | - | -0.289 | 0.5685 | 0.523 | 0.4086 | 0.4874 |  |
| Total FM% | - | - | -0.252 | 0.2096 | -0.368 | 0.1724 | 0.1305 | - | - | -0.261 | 0.1346 | -0.598 | 0.0069 | 0.0069 | - | - | -0.799 | 0.1085 | 0.126 | 0.8391 | 0.9894 |  |
| Arms FM% | - | - | 0.065 | 0.3630 | 0.009 | 0.9255 | 0.7324 | - | - | -0.072 | 0.2491 | -0.129 | 0.1024 | 0.0926 | - | - | 0.064 | 0.6424 | -0.125 | 0.4678 | 0.5385 |  |
| Trunk FM% | - | - | -0.181 | 0.4635 | -0.325 | 0.3259 | 0.2986 | - | - | -0.284 | 0.1372 | -0.813 | 0.0008 | 0.0010 | - | - | -0.306 | 0.4811 | -0.512 | 0.3451 | 0.3346 |  |
| Android FM% | - | - | -0.027 | 0.6947 | -0.022 | 0.8122 | 0.7537 | - | - | -0.096 | 0.0885 | -0.234 | 0.0010 | 0.0011 | - | - | 0.015 | 0.9070 | -0.086 | 0.5774 | 0.6154 |  |
| AOI | - | - | -0.010 | 0.2372 | -0.009 | 0.4202 | 0.3073 | - | - | -0.012 | 0.0611 | -0.024 | 0.0039 | 0.0033 | - | - | 0.003 | 0.7995 | -0.005 | 0.7638 | 0.8050 |  |
| Gynoid FM% | - | - | 0.091 | 0.4514 | 0.143 | 0.3783 | 0.3371 | - | - | 0.187 | 0.0561 | 0.489 | <0.0001 | <0.0001 | - | - | 0.105 | 0.6383 | 0.257 | 0.3563 | 0.3601 |  |
| Legs FM% | - | - | 0.123 | 0.6074 | 0.345 | 0.2827 | 0.2922 | - | - | 0.332 | 0.0796 | 0.902 | 0.0002 | 0.0002 | - | - | 0.143 | 0.7332 | 0.546 | 0.2966 | 0.3117 |  |

Models were adjusted for study cohort, age, sex, marital status, smoking, drinking, type of meal, physical activity level, sleep quality and body mass index.

Abbreviations: WC, waist circumference; FM%, fat mass percentage; AOI, android-to-dynoid fat mass ratio.

β**^#^**: Unstandardized regression coefficient.

*P**: Trend tests among participants in three eating speed subgroups among different education levels.

**Table S7. Associations between eating speed and body fat distribution among different smoking status**

| **Variables** | **Nonsmoker (n=4001)** | | | | | | | **Smoker (n=769)** | | | | | | |
| --- | --- | --- | --- | --- | --- | --- | --- | --- | --- | --- | --- | --- | --- | --- |
|  | **Meal duration<10 min (n=1265)** | | **10≤meal duration<20 min (n=2013)** | | **Meal duration≥20 min (n=723)** | | ***P** for trend** | **Meal duration<10 min (n=304)** | | **10≤meal duration<20 min (n=315)** | | **Meal duration≥20 min (n=150)** | | ***P** for trend** |
|  | **β^#^** | ***P*** | **β^#^** | ***P*** | **β^#^** | ***P*** |  | **β^#^** | ***P*** | **β^#^** | ***P*** | **β^#^** | ***P*** |  |
| WC | - | - | -0.379 | 0.0213 | -0.521 | 0.0153 | 0.0084 | - | - | -0.311 | 0.3488 | 0.052 | 0.9005 | 0.8881 |
| Total FM% | - | - | -0.298 | 0.0332 | -0.413 | 0.0235 | 0.0141 | - | - | -0.456 | 0.1632 | -0.444 | 0.2788 | 0.1913 |
| Arms FM% | - | - | 0.003 | 0.9519 | -0.088 | 0.1741 | 0.2398 | - | - | -0.008 | 0.9351 | -0.064 | 0.6056 | 0.6395 |
| Trunk FM% | - | - | -0.258 | 0.1055 | -0.755 | 0.0003 | 0.0004 | - | - | -0.521 | 0.1126 | -0.127 | 0.7569 | 0.4911 |
| Android FM% | - | - | -0.073 | 0.1099 | -0.192 | 0.0013 | 0.0015 | - | - | -0.008 | 0.9365 | 0.076 | 0.5370 | 0.6080 |
| AOI | - | - | -0.011 | 0.0419 | -0.021 | 0.0020 | 0.0016 | - | - | -0.014 | 0.2565 | -0.003 | 0.8638 | 0.6530 |
| Gynoid FM% | - | - | 0.144 | 0.0710 | 0.417 | <0.0001 | <0.0001 | - | - | 0.228 | 0.1685 | 0.038 | 0.8541 | 0.5999 |
| Legs FM% | - | - | 0.227 | 0.1538 | 0.817 | <0.0001 | 0.0002 | - | - | 0.493 | 0.0848 | 0.166 | 0.6440 | 0.3916 |

Models were adjusted for study cohort, age, sex, education level, marital status, drinking, type of meal, physical activity level, sleep quality and body mass index.

Abbreviations: WC, waist circumference; FM%, fat mass percentage; AOI, android-to-dynoid fat mass ratio.

β**^#^**: Unstandardized regression coefficient.

*P**: Trend tests among participants in three eating speed subgroups in different smoking status.

**Table S8. Associations between eating speed and body fat distribution among different drinking status**

| **Variables** | **Nondrinker (n=2639)** | | | | | | | **Drinker (n=2131)** | | | | | | | | |
| --- | --- | --- | --- | --- | --- | --- | --- | --- | --- | --- | --- | --- | --- | --- | --- | --- |
|  | **Meal duration<10 min (n=881)** | | **10≤meal duration<20 min (n=1372)** | | **Meal duration≥20 min (n=386)** | | ***P** for trend** | **Meal duration<10 min (n=688)** | | **10≤meal duration<20 min (n=956)** | | **Meal duration≥20 min (n=487)** | | | ***P** for trend** | |
|  | **β^#^** | ***P*** | **β^#^** | ***P*** | **β^#^** | ***P*** |  | **β^#^** | ***P*** | **β^#^** | ***P*** | **β^#^** | ***P*** |  | |  |
| WC | - | - | -0.236 | 0.2318 | -0.436 | 0.1175 | 0.0966 | - | - | -0.495 | 0.0269 | -0.404 | 0.1271 | 0.0868 | |  |
| Total FM% | - | - | -0.130 | 0.4399 | -0.068 | 0.7746 | 0.6320 | - | - | -0.567 | 0.0042 | -0.708 | 0.0025 | 0.0014 | |  |
| Arms FM% | - | - | 0.023 | 0.7027 | -0.123 | 0.1432 | 0.2801 | - | - | -0.039 | 0.5540 | -0.069 | 0.3782 | 0.3697 | |  |
| Trunk FM% | - | - | -0.345 | 0.0742 | -0.849 | 0.0018 | 0.0018 | - | - | -0.272 | 0.2067 | -0.516 | 0.0434 | 0.0410 | |  |
| Android FM% | - | - | -0.071 | 0.2002 | -0.205 | 0.0091 | 0.0109 | - | - | -0.056 | 0.3689 | -0.108 | 0.1432 | 0.1392 | |  |
| AOI | - | - | -0.011 | 0.0958 | -0.025 | 0.0045 | 0.0043 | - | - | -0.012 | 0.1105 | -0.012 | 0.1798 | 0.1445 | |  |
| Gynoid FM% | - | - | 0.114 | 0.2418 | 0.401 | 0.0035 | 0.0057 | - | - | 0.219 | 0.0395 | 0.340 | 0.0071 | 0.0055 | |  |
| Legs FM% | - | - | 0.301 | 0.1207 | 0.969 | 0.0004 | 0.0007 | - | - | 0.270 | 0.1883 | 0.552 | 0.0231 | 0.0224 | |  |

Models were adjusted for study cohort, age, sex, education level, marital status, smoking, type of meal, physical activity level, sleep quality and body mass index.

Abbreviations: WC, waist circumference; FM%, fat mass percentage; AOI, android-to-dynoid fat mass ratio.

β**^#^**: Unstandardized regression coefficient.

*P**: Trend tests among participants in three eating speed subgroups in different drinking status.

**Table S9. Associations between eating speed and body fat distribution between different sexes**

| **Variables** | **Men (n=1820)** | | | | | | | **Women (n=2950)** | | | | | | |
| --- | --- | --- | --- | --- | --- | --- | --- | --- | --- | --- | --- | --- | --- | --- |
|  | **Meal duration<10 min (n=644)** | | **10≤meal duration<20 min (n=830)** | | **Meal duration≥20 min (n=346)** | | ***P** for trend** | **Meal duration<10 min (n=925)** | | **10≤meal duration<20 min (n=1498)** | | **Meal duration≥20 min (n=527)** | | ***P** for trend** |
|  | **β^#^** | ***P*** | **β^#^** | ***P*** | **β^#^** | ***P*** |  | **β^#^** | ***P*** | **β^#^** | ***P*** | **β^#^** | ***P*** |  |
| WC | - | - | -0.298 | 0.1814 | -0.408 | 0.1549 | 0.1181 | - | - | -0.404 | 0.0378 | -0.416 | 0.1014 | 0.0580 |
| Total FM% | - | - | -0.476 | 0.0260 | -0.720 | 0.0090 | 0.0049 | - | - | -0.244 | 0.1253 | -0.223 | 0.2823 | 0.1997 |
| Arms FM% | - | - | -0.043 | 0.5137 | -0.077 | 0.3614 | 0.3408 | - | - | 0.037 | 0.5286 | -0.043 | 0.5738 | 0.7261 |
| Trunk FM% | - | - | -0.551 | 0.0128 | -0.596 | 0.0364 | 0.0152 | - | - | -0.165 | 0.3732 | -0.611 | 0.0115 | 0.0160 |
| Android FM% | - | - | -0.074 | 0.2601 | -0.150 | 0.0775 | 0.0706 | - | - | -0.046 | 0.3750 | -0.103 | 0.1331 | 0.1307 |
| AOI | - | - | -0.019 | 0.0219 | -0.025 | 0.0204 | 0.0100 | - | - | -0.006 | 0.2856 | -0.010 | 0.1763 | 0.1554 |
| Gynoid FM% | - | - | 0.302 | 0.0068 | 0.351 | 0.0146 | 0.0053 | - | - | 0.061 | 0.5092 | 0.309 | 0.0106 | 0.0173 |
| Legs FM% | - | - | 0.546 | 0.0055 | 0.644 | 0.0111 | 0.0039 | - | - | 0.104 | 0.5832 | 0.641 | 0.0099 | 0.0175 |

Models were adjusted for study cohort, age, marital status, education level, smoking, drinking, type of meal, physical activity level, sleep quality and body mass index.

Abbreviations: WC, waist circumference; FM%, fat mass percentage; AOI, android-to-dynoid fat mass ratio.

β**^#^**: Unstandardized regression coefficient.

*P**: Trend tests among participants in three eating speed subgroups in different marital status.

**Table S10. Associations between eating speed and body fat distribution among different physical activity levels**

| **Variables** | **Low physical activity level (n=1470)** | | | | | | | **Middle physical activity level (n=2001)** | | | | | | | **High physical activity level (n=1299)** | | | | | | | |
| --- | --- | --- | --- | --- | --- | --- | --- | --- | --- | --- | --- | --- | --- | --- | --- | --- | --- | --- | --- | --- | --- | --- |
|  | **Meal duration<10 min (n=532)** | | **10≤meal duration<20 min (n=679)** | | **Meal duration≥20 min (n=259)** | | ***P** for trend** | **Meal duration<10 min (n=598)** | | **10≤meal duration<20 min (n=1051)** | | **Meal duration≥20 min (n=352)** | | ***P** for trend** | **Meal duration <10 min (n=439)** | | **10≤meal duration<20 min (n=598)** | | **Meal duration≥20 min (n=262)** | | ***P** for trend** |  |
|  | **β^#^** | ***P*** | **β^#^** | ***P*** | **β^#^** | ***P*** |  | **β^#^** | ***P*** | **β^#^** | ***P*** | **β^#^** | ***P*** |  | **β^#^** | ***P*** | **β^#^** | ***P*** | **β^#^** | ***P*** |  |  |
| WC | - | - | -0.178 | 0.5037 | -0.189 | 0.5875 | 0.5212 | - | - | -0.492 | 0.0293 | -0.648 | 0.0284 | 0.0167 | - | - | -0.333 | 0.2527 | -0.257 | 0.4828 | 0.3892 |  |
| Total FM% | - | - | -0.165 | 0.4567 | -0.278 | 0.3365 | 0.3076 | - | - | -0.483 | 0.0187 | -0.737 | 0.0061 | 0.0036 | - | - | -0.143 | 0.5602 | -0.076 | 0.8062 | 0.7344 |  |
| Arms FM% | - | - | 0.047 | 0.5416 | -0.077 | 0.4480 | 0.6252 | - | - | -0.063 | 0.3782 | -0.142 | 0.1306 | 0.1294 | - | - | 0.020 | 0.8127 | -0.039 | 0.7055 | 0.7752 |  |
| Trunk FM% | - | - | 0.010 | 0.9710 | -0.551 | 0.1125 | 0.1771 | - | - | -0.371 | 0.0922 | -0.717 | 0.0129 | 0.0110 | - | - | -0.540 | 0.0497 | -0.637 | 0.0655 | 0.0411 |  |
| Android FM% | - | - | 0.073 | 0.3293 | 0.004 | 0.9711 | 0.7727 | - | - | -0.112 | 0.0803 | -0.221 | 0.0088 | 0.0075 | - | - | -0.165 | 0.0380 | -0.201 | 0.0442 | 0.0267 |  |
| AOI | - | - | 0.004 | 0.6160 | -0.009 | 0.4502 | 0.6071 | - | - | -0.017 | 0.0225 | -0.018 | 0.0689 | 0.0368 | - | - | -0.018 | 0.0614 | -0.023 | 0.0514 | 0.0342 |  |
| Gynoid FM% | - | - | -0.029 | 0.8211 | 0.279 | 0.1029 | 0.1812 | - | - | 0.250 | 0.0268 | 0.401 | 0.0065 | 0.0042 | - | - | 0.201 | 0.1385 | 0.320 | 0.0604 | 0.0484 |  |
| Legs FM% | - | - | -0.059 | 0.8194 | 0.617 | 0.0677 | 0.1326 | - | - | 0.404 | 0.0667 | 0.807 | 0.0051 | 0.0044 | - | - | 0.484 | 0.0680 | 0.687 | 0.0395 | 0.0273 |  |

Models were adjusted for study cohort, age, sex, marital status, education level, smoking, drinking, type of meal, sleep quality and body mass index.

Abbreviations: WC, waist circumference; FM%, fat mass percentage; AOI, android-to-dynoid fat mass ratio.

β**^#^**: Unstandardized regression coefficient.

*P**: Trend tests among participants in three eating speed subgroups in different marital status.

**Table S11. Associations between eating speed and body fat distribution among different types of meal**

| **Variables** | **Meat-based (n=372)** | | | | | | | **Balanced (n=3126)** | | | | | | | **Vegetable-based(n=1272)** | | | | | | | |
| --- | --- | --- | --- | --- | --- | --- | --- | --- | --- | --- | --- | --- | --- | --- | --- | --- | --- | --- | --- | --- | --- | --- |
|  | **Meal duration<10 min (n=119)** | | **10≤meal duration<20 min (n=161)** | | **Meal duration≥20 min (n=92)** | | ***P** for trend** | **Meal duration<10 min (n=990)** | | **10≤meal duration<20 min (n=1570)** | | **Meal duration≥20 min (n=566)** | | ***P** for trend** | **Meal duration <10 min (n=460)** | | **10≤meal duration<20 min (n=597)** | | **Meal duration≥20 min (n=215)** | | ***P** for trend** |  |
|  | **β^#^** | ***P*** | **β^#^** | ***P*** | **β^#^** | ***P*** |  | **β^#^** | ***P*** | **β^#^** | ***P*** | **β^#^** | ***P*** |  | **β^#^** | ***P*** | **β^#^** | ***P*** | **β^#^** | ***P*** |  |  |
| WC | - | - | -0.513 | 0.3368 | -1.278 | 0.0366 | 0.0379 | - | - | -0.214 | 0.2490 | -0.217 | 0.3684 | 0.2974 | - | - | -0.642 | 0.0199 | -0.592 | 0.1088 | 0.0434 |  |
| Total FM% | - | - | -0.676 | 0.1721 | -1.145 | 0.0431 | 0.0391 | - | - | -0.362 | 0.0233 | -0.393 | 0.0580 | 0.0307 | - | - | -0.109 | 0.6522 | -0.258 | 0.4271 | 0.4241 |  |
| Arms FM% | - | - | -0.046 | 0.7725 | -0.354 | 0.0500 | 0.0615 | - | - | 0.052 | 0.3397 | -0.054 | 0.4480 | 0.6491 | - | - | -0.150 | 0.0872 | -0.106 | 0.3686 | 0.2117 |  |
| Trunk FM% | - | - | -0.341 | 0.4957 | -0.920 | 0.1079 | 0.1118 | - | - | -0.374 | 0.0340 | -0.510 | 0.0258 | 0.0155 | - | - | -0.126 | 0.6625 | -0.967 | 0.0122 | 0.0253 |  |
| Android FM% | - | - | -0.119 | 0.4139 | -0.343 | 0.0404 | 0.0433 | - | - | -0.070 | 0.1661 | -0.103 | 0.1185 | 0.0943 | - | - | -0.038 | 0.6421 | -0.202 | 0.0671 | 0.0934 |  |
| AOI | - | - | -0.014 | 0.4287 | -0.032 | 0.1064 | 0.1073 | - | - | -0.012 | 0.0502 | -0.011 | 0.1760 | 0.1059 | - | - | -0.007 | 0.4473 | -0.029 | 0.0237 | 0.0335 |  |
| Gynoid FM% | - | - | 0.257 | 0.2793 | 0.559 | 0.0402 | 0.0398 | - | - | 0.160 | 0.0735 | 0.233 | 0.0444 | 0.0311 | - | - | 0.114 | 0.4212 | 0.580 | 0.0024 | 0.0053 |  |
| Legs FM% | - | - | 0.329 | 0.4862 | 1.226 | 0.0236 | 0.0275 | - | - | 0.297 | 0.0850 | 0.552 | 0.0136 | 0.0110 | - | - | 0.254 | 0.3762 | 1.025 | 0.0078 | 0.0126 |  |

Models were adjusted for study cohort, age, sex, marital status, education level, smoking, drinking, physical activity level, sleep quality and body mass index.

Abbreviations: WC, waist circumference; FM%, fat mass percentage; AOI, android-to-dynoid fat mass ratio.

β**^#^**: Unstandardized regression coefficient.

*P**: Trend tests among participants in three eating speed subgroups in different marital status.

**Table S12. Associations between eating speed and body fat distribution among participants from different areas**

| **Variables** | **Urban (n=3095)** | | | | | | | **Rural (n=1675)** | | | | | | |
| --- | --- | --- | --- | --- | --- | --- | --- | --- | --- | --- | --- | --- | --- | --- |
|  | **Meal duration<10 min (n=946)** | | **10≤meal duration<20 min (n=1574)** | | **Meal duration≥20 min (n=575)** | | ***P** for trend** | **Meal duration<10 min (n=623)** | | **10≤meal duration<20 min (n=754)** | | **Meal duration≥20 min (n=298)** | | ***P** for trend** |
|  | **β^#^** | ***P*** | **β^#^** | ***P*** | **β^#^** | ***P*** |  | **β^#^** | ***P*** | **β^#^** | ***P*** | **β^#^** | ***P*** |  |
| WC | - | - | -0.321 | 0.0729 | -0.412 | 0.0735 | 0.0510 | - | - | -0.322 | 0.2157 | -0.399 | 0.2405 | 0.1804 |
| Total FM% | - | - | -0.402 | 0.0127 | -0.387 | 0.0621 | 0.0306 | - | - | -0.070 | 0.7407 | -0.515 | 0.0642 | 0.0946 |
| Arms FM% | - | - | -0.046 | 0.4132 | -0.127 | 0.0811 | 0.0871 | - | - | 0.074 | 0.3060 | -0.015 | 0.8772 | 0.8837 |
| Trunk FM% | - | - | -0.222 | 0.2095 | -0.647 | 0.0045 | 0.0056 | - | - | -0.368 | 0.1403 | -0.685 | 0.0353 | 0.0279 |
| Android FM% | - | - | -0.072 | 0.1611 | -0.145 | 0.0275 | 0.0253 | - | - | -0.049 | 0.4955 | -0.146 | 0.1179 | 0.1281 |
| AOI | - | - | -0.007 | 0.2192 | -0.012 | 0.1248 | 0.1073 | - | - | -0.015 | 0.0865 | -0.025 | 0.0219 | 0.0154 |
| Gynoid FM% | - | - | 0.124 | 0.1648 | 0.341 | 0.0031 | 0.0037 | - | - | 0.182 | 0.1373 | 0.360 | 0.0250 | 0.0204 |
| Legs FM% | - | - | 0.232 | 0.1868 | 0.728 | 0.0013 | 0.0018 | - | - | 0.293 | 0.2219 | 0.716 | 0.0224 | 0.0224 |

Models were adjusted for age, sex, marital status, education level, smoking, drinking, type of meal, physical activity level, sleep quality and body mass index.

Abbreviations: WC, waist circumference; FM%, fat mass percentage; AOI, android-to-dynoid fat mass ratio.

β**^#^**: Unstandardized regression coefficient.

*P**: Trend tests among participants in three eating speed subgroups in different marital status.

**Table S13. Associations between eating speed and body fat distribution among participants with different sleep quality**

| **Variables** | **Normal sleeper (PSQI<7, n=3414)** | | | | | | | **Poor sleeper (PSQI≥7, n=1356)** | | | | | | | |
| --- | --- | --- | --- | --- | --- | --- | --- | --- | --- | --- | --- | --- | --- | --- | --- |
|  | **Meal duration<10 min (n=1084)** | | **10≤meal duration<20 min (n=1706)** | | **Meal duration≥20 min (n=624)** | | ***P** for trend** | **Meal duration<10 min (n=485)** | | **10≤meal duration<20 min (n=622)** | | **Meal duration≥20 min (n=249)** | | ***P** for trend** |  |
|  | **β^#^** | ***P*** | **β^#^** | ***P*** | **β^#^** | ***P*** |  | **β^#^** | ***P*** | **β^#^** | ***P*** | **β^#^** | ***P*** |  |  |
| WC | - | - | -0.461 | 0.0092 | -0.542 | 0.0182 | 0.0081 | - | - | -0.130 | 0.6302 | -0.072 | 0.8376 | 0.7656 |  |
| Total FM% | - | - | -0.481 | 0.0017 | -0.303 | 0.1262 | 0.0418 | - | - | 0.141 | 0.5530 | -0.692 | 0.0248 | 0.0738 |  |
| Arms FM% | - | - | -0.005 | 0.9249 | -0.098 | 0.1559 | 0.2063 | - | - | -0.020 | 0.8107 | -0.086 | 0.4181 | 0.4472 |  |
| Trunk FM% | - | - | -0.434 | 0.0103 | -0.889 | <0.0001 | <0.0001 | - | - | -0.007 | 0.9797 | -0.129 | 0.7159 | 0.7483 |  |
| Android FM% | - | - | -0.102 | 0.0359 | -0.239 | 0.0002 | 0.0002 | - | - | 0.008 | 0.9163 | 0.066 | 0.5143 | 0.5552 |  |
| AOI | - | - | -0.014 | 0.0138 | -0.025 | 0.0007 | 0.0004 | - | - | -0.003 | 0.7145 | 0.002 | 0.8505 | 0.9435 |  |
| Gynoid FM% | - | - | 0.223 | 0.0085 | 0.448 | <0.0001 | <0.0001 | - | - | 0.008 | 0.9550 | 0.131 | 0.4620 | 0.5158 |  |
| Legs FM% | - | - | 0.401 | 0.0157 | 0.974 | <0.0001 | <0.0001 | - | - | 0.039 | 0.8849 | 0.176 | 0.6143 | 0.6370 |  |

Models were adjusted for study cohort, age, sex, marital status, education level, smoking, drinking, type of meal, physical activity level and body mass index.

Abbreviations: PSQI, the Pittsburgh Sleep Quality Index; WC, waist circumference; FM%, fat mass percentage; AOI, android-to-dynoid fat mass ratio.

β**^#^**: Unstandardized regression coefficient.

*P**: Trend tests among participants in three eating speed subgroups in different marital status.

**Table S14. Prevalence of chronic diseases among different age groups (n=4770)**

| **Variables** | **Age groups** | | | ***P**** |
| --- | --- | --- | --- | --- |
|  | **18**≤**age<45 (n=1123)** | **45**≤**age<65 (n=2681)** | **65**≤**age≤80 (n=966)** |  |
| Cardiovascular metabolic diseases, n(%) | 192 (17.1) § | 1006 (37.52) # | 480 (49.69) † | <0.0001 |
| Osteoarticular diseases, n(%) | 42 (3.74) § | 370 (13.8) # | 186 (19.25) † | <0.0001 |
| Cancers, n(%) | 14 (1.25) § | 86 (3.21) # | 32 (3.31) † | 0.0018 |

Data are n (%) unless otherwise indicated.

*P**: Chi-square tests among participants in three groups.

§: *P*<0.05, significant difference between participants in group 1 and group 2.

#: *P*<0.05, significant difference between participants in group 2 and group 3.

†: *P*<0.05, significant difference between participants in group 1 and group 3.

**Table S15. Associations between eating speed and body fat distribution among different age groups (additionally adjusted for** **chronic diseases)**

| **Variables** | **18**≤**age<45 (n=1123)** | | | | | | | **45**≤**age<65 (n=2681)** | | | | | | | | **65**≤**age≤80 (n=966)** | | | | | | | | |
| --- | --- | --- | --- | --- | --- | --- | --- | --- | --- | --- | --- | --- | --- | --- | --- | --- | --- | --- | --- | --- | --- | --- | --- | --- |
|  | **Meal duration<10 min (n=336)** | | **10≤meal duration<20 min (n=567)** | | **Meal duration≥20 min (n=220)** | | ***P** for trend** | **Meal duration<10 min (n=945)** | | **10≤meal duration<20 min (n=1298)** | | **Meal duration≥20 min (n=438)** | | ***P** for trend** | **Meal duration <10 min (n=288)** | | | **10≤meal duration<20 min (n=463)** | | **Meal duration≥20 min (n=215)** | | ***P** for trend** | |  |
|  | **β^#^** | ***P*** | **β^#^** | ***P*** | **β^#^** | ***P*** |  | **β^#^** | ***P*** | **β^#^** | ***P*** | **β^#^** | ***P*** |  | **β^#^** | | ***P*** | **β^#^** | ***P*** | **β^#^** | ***P*** |  |  |  |
| WC | - | - | -0.344 | 0.2614 | -0.850 | 0.0261 | 0.0275 | - | - | -0.526 | 0.0058 | -0.194 | 0.4556 | 0.1467 | - | | - | 0.383 | 0.2670 | 0.122 | 0.7690 | 0.6847 |  |  |
| Total FM% | - | - | -0.418 | 0.1417 | -0.602 | 0.0894 | 0.0758 | - | - | -0.304 | 0.0641 | -0.512 | 0.0221 | 0.0137 | - | | - | -0.233 | 0.4301 | -0.071 | 0.8427 | 0.7802 |  |  |
| Arms FM% | - | - | 0.014 | 0.8748 | -0.139 | 0.1974 | 0.2483 | - | - | -0.001 | 0.9899 | 0.018 | 0.8222 | 0.8543 | - | | - | -0.016 | 0.8791 | -0.114 | 0.3781 | 0.4002 |  |  |
| Trunk FM% | - | - | -0.323 | 0.2340 | -0.708 | 0.0369 | 0.0367 | - | - | -0.246 | 0.1685 | -0.367 | 0.1327 | 0.0952 | - | | - | -0.093 | 0.8066 | -0.416 | 0.3646 | 0.3811 |  |  |
| Android FM% | - | - | -0.084 | 0.2860 | -0.281 | 0.0043 | 0.0056 | - | - | -0.084 | 0.1007 | -0.012 | 0.8668 | 0.5263 | - | | - | 0.048 | 0.6627 | -0.100 | 0.4540 | 0.5175 |  |  |
| AOI | - | - | -0.015 | 0.0633 | -0.032 | 0.0017 | 0.0017 | - | - | -0.011 | 0.0838 | -0.008 | 0.3579 | 0.1981 | - | | - | 0.002 | 0.9016 | -0.006 | 0.7004 | 0.7303 |  |  |
| Gynoid FM% | - | - | 0.251 | 0.0720 | 0.605 | 0.0005 | 0.0006 | - | - | 0.135 | 0.1354 | 0.202 | 0.1018 | 0.0696 | - | | - | -0.003 | 0.9850 | 0.090 | 0.6870 | 0.7083 |  |  |
| Legs FM% | - | - | 0.308 | 0.2565 | 0.847 | 0.0126 | 0.0142 | - | - | 0.213 | 0.2261 | 0.315 | 0.1880 | 0.1436 | - | | - | 0.112 | 0.7559 | 0.570 | 0.1896 | 0.2069 |  |  |

Models were adjusted for chronic diseases (including cardiovascular diseases, metabolic diseases, osteoarticular diseases and cancers), study cohort, age, sex, education level, marital status, smoking, drinking, type of meal, physical activity level, sleep quality and body mass index.

Abbreviations: WC, waist circumference; FM%, fat mass percentage; AOI, android-to-dynoid fat mass ratio.

β**^#^**: Unstandardized regression coefficient.

*P**: Trend tests among participants in three eating speed subgroups in each age group.
